# Challenges of Cushing's syndrome and bariatric surgery: a case report with literature review

**DOI:** 10.1093/jscr/rjaf366

**Published:** 2025-06-08

**Authors:** Shaho F Ahmed, Sherzad W Mahmood, Deari A Ismaeil, Hiwa O Baba, Karzan M Salih, Soran H Tahir, Hemn H Kaka Ali, Rebaz O Mohammed, Swara H Abdullah, Rekan K Hama Ali, Berun A Abdalla, Fahmi H Kakamad

**Affiliations:** Scientific Affairs Department, Smart Health Tower, Madam Mitterrand Street, Sulaymaniyah 46001, Iraq; Department of Medicine, Shar Hospital, Malik Mahmud Ring Road, Sulaymaniyah 46001, Iraq; Scientific Affairs Department, Smart Health Tower, Madam Mitterrand Street, Sulaymaniyah 46001, Iraq; Scientific Affairs Department, Smart Health Tower, Madam Mitterrand Street, Sulaymaniyah 46001, Iraq; College of Medicine, University of Sulaimani, Madam Mitterrand Street, Sulaymaniyah 46001, Iraq; Scientific Affairs Department, Smart Health Tower, Madam Mitterrand Street, Sulaymaniyah 46001, Iraq; Scientific Affairs Department, Smart Health Tower, Madam Mitterrand Street, Sulaymaniyah 46001, Iraq; Scientific Affairs Department, Smart Health Tower, Madam Mitterrand Street, Sulaymaniyah 46001, Iraq; College of Medicine, University of Sulaimani, Madam Mitterrand Street, Sulaymaniyah 46001, Iraq; Scientific Affairs Department, Smart Health Tower, Madam Mitterrand Street, Sulaymaniyah 46001, Iraq; Gastroenterology and Hepatology Department, Sulaimani Teaching Hospital, Zanko Street, Sulaymaniyah 46001, Iraq; Scientific Affairs Department, Smart Health Tower, Madam Mitterrand Street, Sulaymaniyah 46001, Iraq; Department of Medicine, Shar Hospital, Malik Mahmud Ring Road, Sulaymaniyah 46001, Iraq; Scientific Affairs Department, Smart Health Tower, Madam Mitterrand Street, Sulaymaniyah 46001, Iraq; Scientific Affairs Department, Smart Health Tower, Madam Mitterrand Street, Sulaymaniyah 46001, Iraq; Kscien Organization for Scientific Research (Middle East Office), Hamdi Street, Azadi Mall, Sulaymaniyah 46001, Iraq; Scientific Affairs Department, Smart Health Tower, Madam Mitterrand Street, Sulaymaniyah 46001, Iraq; College of Medicine, University of Sulaimani, Madam Mitterrand Street, Sulaymaniyah 46001, Iraq; Kscien Organization for Scientific Research (Middle East Office), Hamdi Street, Azadi Mall, Sulaymaniyah 46001, Iraq

**Keywords:** hypercortisolism, adrenocorticotropic hormone, Cushing syndrome, obesity, bariatric surgery

## Abstract

Cushing’s disease (CD), caused by an adrenocorticotropic hormone-secreting pituitary adenoma, is challenging to diagnose, especially in obese patients post-bariatric surgery. This report discusses a misdiagnosed case of CD in a 42-year-old obese male with hypertension. CD was suspected only after surgery, confirmed by magnetic resonance imaging (MRI) showing a pituitary macroadenoma. Despite transsphenoidal surgery and ketoconazole therapy, the patient suffered liver failure and died. Among 20 CD reviewed cases in the literature, 65% were misdiagnosed. MRI and immunohistochemistry confirmed tumors, with 55% achieving remission post-surgery. Screening for CD before bariatric surgery may prevent mismanagement and complications.

## Introduction

Cushing syndrome (CS) is an endocrine disorder caused by excessive cortisol from adrenal or pituitary neoplasms or prolonged corticosteroid use [1]. Endogenous CS affects 0.2–5.0 cases per million annually, with a prevalence of 39–79 cases per million. Adrenocorticotropic hormone-dependent CS accounts for 80%–85% of cases [2]. CS presents with hypertension, diabetes, osteoporosis, obesity, psychiatric disorders, cardiovascular diseases, and thrombosis [3, 4]. Bariatric surgery (BS) has risen globally, improving obesity-related conditions [5, 6]. However, undiagnosed CS in BS patients may worsen outcomes [7, 8].

The references have been confirmed as eligible [9]. The report is structured according to CaReL criteria and includes a summary of the relevant literature [10].

This report aims to describe an obese patient who has undergone bariatric surgery with an undiagnosed CS.

## Case presentation

### Patient information

A 42-year-old male with a body mass index (BMI) of 45.0 and a history of hypertension, presented with hyperglycemia and increased weakness after one month of bariatric surgery. He had a 40-pack-year smoking history, although had quit smoking 4 years prior.

### Clinical findings

The patient was unable to move properly and required assistance. This weakness was associated with fatigue, joint pain, polyuria, polydipsia, and a good appetite. An abdominal examination revealed a soft, distended, doughy abdomen with multiple striae and a laparoscopic port scar. He had normal hair distribution, bilateral gynecomastia, normal-sized testicles, and multiple pigmented spots on his skin, especially on the knuckles, along with easy bruising and central obesity. An ejection systolic murmur in the aortic area was noted. The patient’s power and reflexes were decreased. Otherwise, all other physical examination findings were normal.

### Diagnostic approach

**Figure 1:**
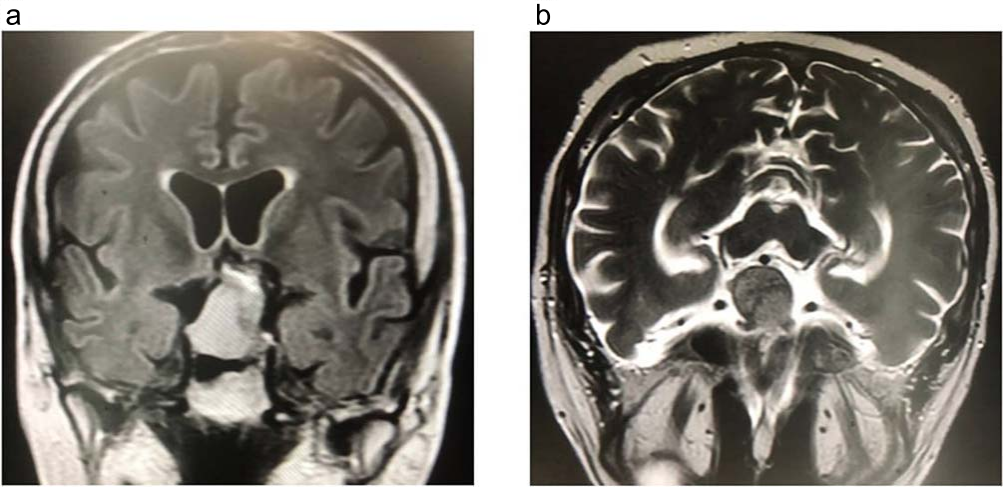
Brain MRI. (a) FLAIR coronal section. (b) T2WI axial section, show pituitary macroadenoma, measuring 35 × 27 × 25 mm. The sellar mass expands the sella and extends into suprasellar region, exhibiting a heterogeneous signal. It is in contact with optic chiasm and partially encases internal carotid artery (ICA).

The preoperative laboratory tests for the patient revealed a white blood cell count (WBC) of 12.7 × 10^9^/L, hemoglobin (HGB) of 16.1 g/dL, glycated hemoglobin (HbA1c) of 6.44%, C-reactive protein (CRP) of 1.87 mg/L, and serum creatinine of 0.99 mg/dL (Table 1). A diagnosis of Cushing's disease (CD) was made based on a significantly elevated adrenocorticotropic hormone (ACTH) level of 318 pg/mL, a markedly elevated cortisol level of 1750 nmol/L, and an abnormal 1 mg dexamethasone suppression test result of 1261 nmol/L. Magnetic resonance imaging (MRI) revealed a pituitary macroadenoma measuring 35 × 27 × 25 mm, located in the sellar region, replacing the pituitary gland, and extending into the suprasellar space. The tumor exhibited a heterogeneous appearance with cystic changes, caused mild compression of the optic chiasm, and resulted in thinning of the sellar floor, abutting nearby structures, including the cavernous sinuses, internal carotid arteries, and posterior cerebral arteries, without evidence of invasion or aggressive features (Fig. 1). Cerebral computed tomography angiography (CTA) revealed the tumor abutted the A1 segments of the anterior cerebral artery anteriorly, the cavernous sinuses and internal carotid arteries laterally, and the basilar tip and posterior cerebral arteries posteriorly, all without encasement. Additionally, the sphenoid sinuses showed complete sellar-type pneumatization.

**Table 1:** Laboratory findings before and after bariatric and transsphenoidal surgery.

**Laboratory Test**					
**Tests**	**Pre-Bariatric Surgery**	**Post Bariatric Surgery**	**Post Transsphenoidal Surgery**	**Normal Range**	**Unit**
WBC	12.7	11.2	6.3	3.5–10	10^9^/L
HGB	16.1	14.7	11.7	11.5–16.5	g/dL
Urea	0.056	28	40	16.6–48.5	mg/dL
S. creatinine	0.99	0.9	1.3	men: 0.7–1.2	mg/dL
TSH	0.056	N/A	N/A	>18 year: 0.4–4.2	ulU/mL
FT3	2.21	N/A	N/A	Adult: 2.0–4.2	pmol/L
FT4	4.5	N/A	N/A	Adults >19 year: 12–22	pmol/L
HbA1c	6.44	8.01	N/A	Normal: 4.0%–5.6%. Pre diabetes: 5.7%–6.4%. Diabetes: 6.5% or higher	%
Chloride (Cl^–^)	101.0	95.8	105	95–115	mmol/L
Potassium (K^+^)	5	2.33	3.12	3.5–5.1	mmol/L
Sodium (Na^+^)	141.1	141.2	139	135–145	mmol/L
Magnesium (Mg)	N/A	1.07	N/A	1.52–2.45	mg/dL
Iron	71.5	N/A	N/A	Men: 65–176	μg/dL
Calcium	8.6	N/A	N/A	Adult 18–60 year: 8.6–10	mg/dL
C-Reactive protein CRP)	1.87	7.9	22.46	<5	mg/L
Total serum bilirubin (TSB)	1.6	N/A	N/A	<0.2	mg/dL
Total serum bilirubin (TSB)-Direct	0.62	N/A	N/A	(Adult and children) <1.2	mg/dL
Vitamin D3	22.3	N/A	N/A	Deficient: <20, Insufficient: 20–29, Sufficient: 30–50, Potentially toxic: >100	ng/mL
Adrenocorticotropic hormone (ACTH)	N/A	318	64.90	7.2–63.6	pg/mL
S. Cortisol	N/A	1750	473.7	Morning: 166–507 Afternoon: 73.8–291	nmol/L
Albumin	N/A	3.06	N/A	>18 years: 3.5–5.2	g/dL
Amylase	N/A	20	N/A	28–120	U/L
Lipase	17	15	N/A	13–60	U/L
Dexamethasone suppression test	N/A	1261	N/A	<50 Dexamethasone 1 mg is administered orally between 11 pm and midnight. Serum cortisol levels are drawn the next morning between 8 and 9 AM	noml/L
Aspartate Aminotransferase (AST)	32	N/A	N/A	Male: <40	lU/L
Alanine Aminotransferase (ALT)	48.8	47	N/A	Male: <50	lU/L
Salmonella Typhoid lgM	N/A	N/A	Positive		
Salmonella Typhoid lgG	N/A	N/A	Negative		
Bleeding time	2.6	N/A	N/A	2–6	Minutes
Prothrombin Time (PT)	12.9	N/A	N/A	11–16	Seconds
INR	0.87	N/A	N/A	Normal: 0.1–1.1 Therapeutic range: Standard intensity warfarin: 2–3 High intensity warfarin: 2.5–3.5	
HIV Ab	Negative				
HBs Ag	Negative				

An abdominal ultrasonography was unremarkable, while an esophago-gastro-duodenoscopy identified multiple anastomotic site erosions and raised erosions in the pre-pyloric region. Coronary CTA showed 40% mid-tubular stenosis in the left anterior descending artery.

### Therapeutic intervention

After intensive care unit stabilization for electrolyte imbalances, the patient underwent transsphenoidal surgery for pituitary macroadenoma. Postoperative tests showed high cortisol and ACTH levels. The patient was readmitted due to lower limb weakness, myopathy, and hypotension, with persistently elevated cortisol. MRI confirmed residual macroadenoma. A second surgery and radiotherapy were considered but deemed challenging, leading to ketoconazole initiation, which initially improved symptoms. However, the patient later developed liver deterioration, marked by elevated liver enzymes, prolonged prothrombin time, low albumin, and high bilirubin.

### Follow-up and outcome

Despite efforts to manage the acute liver failure, the patient's condition worsened, leading to his demise.

## Discussion

CS is an endocrine disorder caused by excess glucocorticoids. It is classified as ACTH-dependent (from pituitary tumors or excess ACTH) or ACTH-independent (from adrenal tumors or glucocorticoid overuse). Pseudo-CS must be ruled out. Normally, ACTH from the pituitary stimulates adrenal cortisol production, but prolonged corticosteroid use suppresses the hypothalamic-pituitary-adrenal (HPA) axis [1, 2]. Among cases reviewed in the literature, 90% were female, with 90% having pituitary adenomas and 10% adrenal adenomas [7, 8, 11, 12] (Table 2). This study involved a male with a pituitary macroadenoma.

**Table 2:** Summary of literature on patients with CD undergoing bariatric surgery.

**Author**	**Age**	**Country**	**Gender**	**BMI**	**Medical history**	**Complication after BS**	**Presentation**	**DX for CS prior BS**	**Treat for CS prior to BS**	**LNSC(nmol/L)**	**UFC** **(μg/dL)**	**ACTH (pg/mL)**	**DST(μg/dL)**	**Histology& IHC**	**Clinical finding (MRI)**	**Management for CS**	**Follow-up**
Javorsky *et al* [7]	30	USA	Female	48 kg/m^2a^	6 out of 11 patients had diabetes mellitus, hypertension was present in 6 out of 11 patients^b^	N/A	N/A	Yes	Yes	N/A	74	N/A	6.3	PT	N/A	Pituitary surgery	Remission
	40		Female			N/A	N/A	Yes	Yes	N/A	187	N/A	N/A	PT was negative by staining	0.5 cm PT	Pituitary surgery	Remission
	27		Female			N/A	N/A	Yes	Yes	N/A	111	N/A	N/A	PT	N/A	Pituitary surgery	Remission
	41		Female			N/A	N/A	Yes	Yes	N/A	155	N/A	N/A	PT	N/A	Pituitary surgery	Adrenal insufficiency
	42		Female			N/A	N/A	Yes	No	5.1	251	N/A	10.9	IPSS positive for pituitary source	N/A	Pituitary surgery	Persistent CS
	31		Female			N/A	N/A	Yes	No	8.7	38	N/A	4.9	PT	N/A	Pituitary surgery	Adrenal insufficiency
	24		Female			N/A	N/A	No	No	9.5	50	N/A	N/A	PT	N/A	Pituitary surgery	Remission
	56		Female			N/A	N/A	No	No	9	109	N/A	10.3	PT	N/A	Pituitary surgery	No recovery, required bilateral adrenalectomy
	41		Female			N/A	N/A	No	No	24.3	59.7	N/A	N/A	PT	N/A	Pituitary surgery	Remission
	25		Female			N/A	N/A	No	No	N/A	N/A	N/A	N/A	PT	N/A	Pituitary surgery	Clinical and biochemical remission with later recurrence.
	22		Female			N/A	N/A	No	No	N/A	517	N/A	17	PT	N/A	Pituitary surgery	Remission
	29		Female			N/A	N/A	No	No	N/A	260	N/A	31	PT	N/A	Pituitary surgery	Remission
	50		Female			N/A	N/A	No	No	775	6502	N/A	N/A	IPSS positive for pituitary source	N/A	Pituitary surgery	Persistent CS after surgery
	26		Female			N/A	N/A	No	No	16.5	114	N/A	16	PT	N/A	Pituitary surgery	N/A
	44		Female			N/A	N/A	No	No	N/A	Elevated	N/A	23	N/A	1.4 cm PT	Pituitary surgery	Remission
	48		Female			N/A	N/A	No	No	5.6	31	N/A	14.1	N/A	3.6 cm left AD	Adrenalectomy	Remission
Borsoi *et al* [8]	49	Austria	Female	61.6 kg/m^2^	Hypertension, Hashimoto’s thyroiditis	N/A	Insomnia, fatigue, extreme skin sensitivity to sunlight, buffalo hump, moon face	Yes	Yes	N/A	177	<5	13	N/A	1.93 cm AT	Unilateral adrenalectomy	Improved
Fleseriu *et al* [11]	50	USA	Female	39 kg/m^2^	Hypertension, hyperglycemia	Nausea, abdominal pain	Facial rounding and plethora	N/A	N/A	N/A	2400	70	N/A	N/A	1.0 cm PT	Two pituitary tumor resection, bilateral adrenalectomy	Improved
	27	USA	Male	53 kg/m^2^	Hypertension, hyperlipidemia	Persistent nausea and abdominal pain, malnutrition	Lower extremity edema, facial plethora, proximal muscle weakness	N/A	N/A	N/A	193	N/A	9.5 (overnight)	N/A	0.2 cm PT	Transsphenoidal	Dead
Pedro *et al* [12]	53	Portugal	Male	45.7 kg/m^2^	Hypertension, chronic pain	osteoporosis	Rib cage pain, difficulty with gait, facial plethora, proximal muscle weakness	No	No	N/A	686.6	46.3	20	N/A	1.46 cm PT	Transsphenoidal	Remission

^a^Mean.

^b^In the study by Javorsky *et al.*, medical history was indicated for 11 out of 16 patients before BS. IPSS, inferior petrosal sinus sampling; CS, Cushing syndrome; ACTH, adrenocorticotropic hormone; DST, dexamethasone suppression test; UFC, urine free cortisol; LNSC, late night salivary cortisol; MRI, magnetic resonance imaging; BMI, body mass index; PT, pituitary tumor; AT, adrenal tumor; Dx, diagnosis.

CS’s most specific features include easy bruising, purple striae, and facial plethora due to elevated cortisol, while nonspecific signs include weight gain, hypertension, and hypokalemia [13]. Reviews by Fleseriu, Borsoi, and Pedro found facial plethora in all CS patients, while proximal muscle weakness appeared in only two cases. All were obese [8, 11, 12]. This patient presented with easy bruising, weakness, pigmented skin, facial plethora, and class III obesity (BMI 45 kg/m^2^). Undiagnosed CS in bariatric patients raises thromboembolic and bone loss risks [8]. Post-surgery, the patient’s HbA1c rose, possibly due to CS activation.

Diagnosing CS in bariatric patients is challenging due to symptom overlap with obesity-related conditions [7]. No single test distinguishes CS from primary obesity [11]. First-line tests include the 1 mg dexamethasone suppression test, 24-h urinary-free cortisol, and late-night salivary cortisol, each with limitations [14]. ACTH levels help differentiate subtypes: high ACTH suggests ACTH-dependent CS, while low ACTH indicates ACTH-independent CS. MRI or inferior petrosal sinus sampling (IPSS) confirms the source [15]. In literature, 60% underwent dexamethasone suppression test (DST), 95% had urinary-free cortisol tests, and 40% had late-night salivary cortisol tests [7, 8, 11, 12]. In this case, the diagnosis was confirmed via DST, ACTH levels, and MRI.

Undiagnosed CS carries severe risks, including a 50% 5-year survival rate in severe cases, a fourfold increase in mortality, and a 20% morbidity and 10% mortality rate in untreated surgical patients, mitigated by anticoagulation. It also contributes to malnutrition and osteoporosis in 40% of patients [11]. Early screening and treatment before BS improve outcomes. Adrenalectomy for CS significantly improves BMI and blood pressure [8].

The primary treatment for endogenous CS is tumor resection. For ACTH-dependent CS, transsphenoidal pituitary surgery has an 80% remission rate, while ACTH-independent cases require adrenalectomy. Medical or radiation therapy is an option for recurrence or ineligible patients [13, 15]. Among 20 cases reviewed in literature, 10% underwent adrenalectomy and 10% had transsphenoidal surgery [7, 8, 11, 12]. In the present case, the patient underwent transsphenoidal surgery. In conclusion, screening for obesity's underlying cause before bariatric surgery may prevent mismanagement and complications.
